# Peripheral follicular cytotoxic T -like cells in Kawasaki disease with coronary artery aneurysms: A case report

**DOI:** 10.1097/MD.0000000000023714

**Published:** 2020-12-24

**Authors:** Meng Xu, Jinxiang Liu, Lu Pan, Sirui Yang

**Affiliations:** Department of Pediatric Rheumatology, Immunology and Allergy, The First Hospital of Jilin University, Changchun, China.

**Keywords:** coronary artery aneurysm, follicular cytotoxic T cells, Kawasaki disease

## Abstract

**Introduction:**

Kawasaki disease (KD) is the leading cause of acquired heart abnormalities during childhood. The infiltration of CD8+ T cells plays an essential role in the formation of coronary aneurysms. Follicular cytotoxic T (Tfc) cells are a newly defined subset of CD8+ T cells that express CXC-chemokine receptor 5. The role of Tfc cells in KD is unclear. However, in this report, we present 2 KD children with sustained coronary artery aneurysms (CAA), and we found that their peripheral C-X-C Chemokine Receptor 5+ T cells contained quite amounts of CD4 negative cells. Importantly, these cells have never been reported in KD.

**Patients concerns:**

Case 1 was a 3-year-old boy with a complaint of continuous fever for 6 days and conjunctival injection for 3 days. Case 2 was a 6-month-old boy who was hospitalized because of persistent fever for 5 days, rashes and conjunctival injection for 1 day.

**Diagnosis:**

Case 1 was diagnosed with KD according to typical symptoms and signs including fever over 5 days, conjunctival injection, rashes, swelling cervical lymph nodes and a strawberry tongue. Case 2 had atypical symptoms including persistent fever for 5 days, rashes and conjunctival injection, and he was diagnosed with KD based on the echocardiographic findings.

**Intervention:**

Both the 2 patients received intravenous immunoglobulin and oral aspirin. Besides, case 1 was given the second infusion of intravenous immunoglobulin, intravenous prednisolone and low-molecular-weight heparin.

**Outcomes:**

The CAA of case 1 did not regress until the 12th month after disease onset. The CAA of patient 2 began to regress at the third month after disease onset. During the months from disease onset to the recent follow-up, no cardiovascular events had occurred.

**Conclusions:**

We speculate that Tfc cells may be associated with the formation of CAA. Further studies with larger sample size and functional analysis of these cells are needed.

## 1 Introduction

Kawasaki disease (KD) is an acute, systemic vasculitis with special predilection for coronary arteries. The status of coronary artery abnormalities can be assessed with luminal dimensions that are detected by echocardiography and then based on the Z score system corrected by body surface area (BSA), the severity of coronary involvement can be classified into no involvement, dilation only, small aneurysm, medium aneurysm, and large aneurysm.^[1]^ In contrast with the patients without coronary involvement, patients with coronary artery aneurysms (CAA) have a higher risk of cardiovascular events such as arrhythmia, myocardial infarction, and even sudden death.^[2]^ The formation of CAA is a complex procedure including the activation of both innate and adaptive immunity. To date, histological investigations have demonstrated that the infiltration of CD8+ T cells into the vessel wall play a critical role in the development of CAA.^[3,4]^ However, the mechanisms have not been well-understood. In this article, we involved 2 KD patients with persistent CAA and found high levels of circulating CXC-chemokine receptor 5 (CXCR5) expressing CD4- T cells.

## 2 Case report

**Figure 1:**
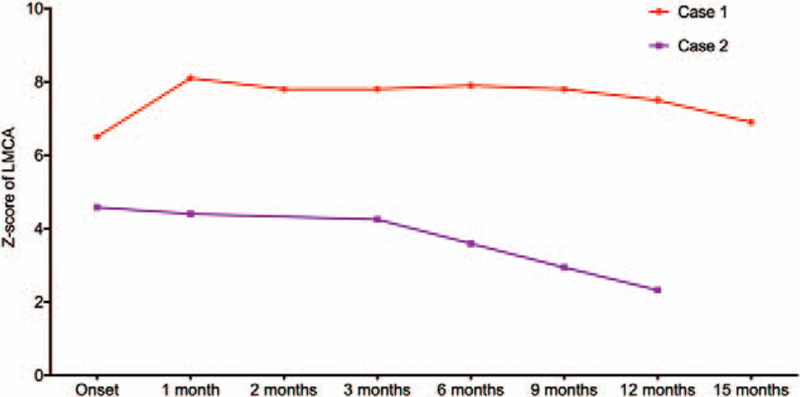
The variation of LMCA-Z-score of the 2 patients from disease onset to the recent follow-up. LMCA, left main coronary artery.

Case 1: A 3-year-old boy was admitted with the complaint of continuous fever for 6 days and conjunctival injection for 3 days on December 4, 2016. Physical examination found diffuse erythematous rashes on the trunk and extremities, bilateral bulbar conjunctival injection without exudate injection, swelling cervical lymph nodes and a strawberry tongue. Laboratory tests showed increased white blood counts and platelets, elevated inflammatory indicators and reduced serum albumin (Table 1). Initial echocardiography indicated the formation of a medium aneurysm in the left main coronary artery (LMCA). Accordingly, the patient was diagnosed with Kawasaki disease. Initial treatment included intravenous immunoglobulins (IVIG) at a dose of 2 g/kg within 1 day, 100 mg/kg/d of oral aspirin in divided doses. However, the patient had persistent fever during the next 36 hours and then he was administrated with the second infusion of IVIG (2 g/kg) and intravenous prednisolone (2 mg/kg/d). After that, his symptoms were improved. Subsequent treatment included low-dose of oral aspirin and a tapered dose of oral prednisolone. One month after the disease onset, echocardiography showed a large aneurysm in LMCA (Z-score<10 but the diameter is 8.1 mm) and thereby low-molecular-weight heparin was administrated additionally. The recent 2 echocardiography indicated that the size of the aneurysm was inclined to regress. The variation of the Z-score was shown in Figure 1. During the months from disease onset to the recent follow-up, no cardiovascular events had occurred.

**Table 1:** The clinical characteristics of the study participants.

	CASE 1	CASE 2	Normal range
WBC, 10^9^/L	14.46	20.38	3.5 – 9.5
Neutrophils, 10^9^/L	11.57	12.3	1.8 – 6.3
lymphocyte, 10^9^/L	1.3	5.6	1.1 – 3.2
Platelet, 10^9^/L	473	328	125 – 350
CRP, mg/L	33	145	0 – 3
ESR, mm/1h	84	98	0 – 20
Procalcitonin, ng/ml	0.36	0.23	0 – 0.5
AST, U/L	38	29	13 – 35
ALT, U/L	22	46	7 – 40
Albumin, g/L	22.7	40.6	40 – 55
IgG, g/L	7.37	4.08	8.6 – 17.4
IgA, g/L	0.83	0.33	1.0 – 4.2
IgM, g/L	1.23	0.28	0.5 – 2.8
C3, g/L	1.61	1.09	0.7 – 1.4
C4, g/L	0.48	0.22	0.1 – 0.4
Blood culture	Negative	Negative	Negative

**Figure 2:**
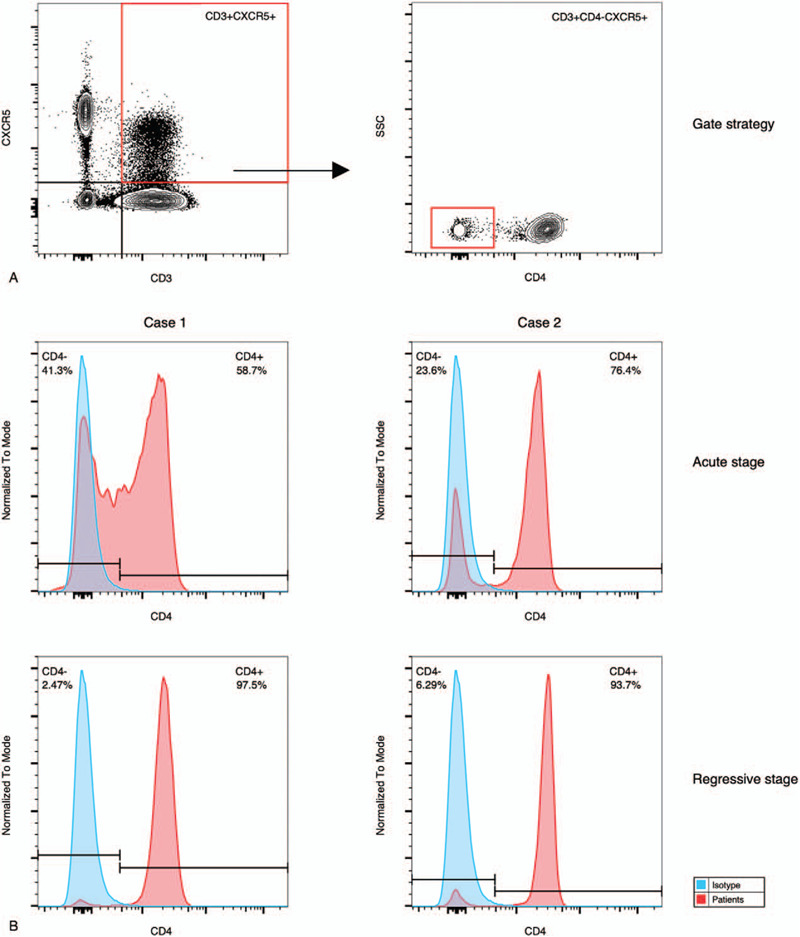
Flow cytometry analysis of the circulating CXCR5+ T cells of the 2 patients. (A) Gate strategy. (B) The percentages of CD4 negative subpopulation of the 2 patients in different stages.

Three milliliters fresh blood were collected at admission and at the time when aneurysm began to regress, respectively. Peripheral blood mononuclear cells were isolated by density-gradient centrifugation, and then stained with fluorescent anti-CD3, anti-CD4, and anti-CXCR5, and analyzed using multicolor flow cytometry. These cells were measured by gating initially live lymphocytes, subsequently CD3+CXCR5+ T cells, and finally CD4 negative population (Fig. 2A). To ensure proper gating strategy, isotype controls were used to determine the gating parameters. As shown in Figure 2B, we found that 41.3% of circulating CXCR5+ T cells were CD4- T cells in the acute stage and 2.47% in the regressive stage.

Case 2: A 6-month-old boy came to our hospital due to persistent fever for 5 days, rashes and conjunctival injection for 1 day on March 17, 2018. There was no definite correlation between fever and rashes. The patient had no medical history from birth. Additional signs necessary for KD diagnosis was not found. The diagnosis was established after the 2-dimensional echocardiography, which showed a small aneurysm in LMCA and another small aneurysm in left anterior descending. Laboratory examination showed significantly increased levels of white blood counts, CRP and ESR, and a slightly elevated level of alanine transaminase. The patient was given only 1 infusion of IVIG (2 g/kg) within 1 day. In addition, 100 mg/kg/d of oral aspirin was given from the establishment of diagnosis to the third day after defervescence and followed by a dose of 5 mg/kg/d. The patient was followed at the 1st, 3rd, 6th, 9th and 12th month after disease onset. At the first follow-up, the echocardiography revealed that the diameter of left anterior descending was normal, whereas the variation of LMCA was not obvious. At the third month after the disease onset, his aneurysm began to regress. The most recent echocardiography performed on April 2, 2019, only showed dilatation of LMCA (Fig. 1). During the past months, no cardiovascular events had occurred. His peripheral blood mononuclear cells were processed in the same method as mentioned in Case 1. We found that 23.6% of CXCR5+ T cells were CD4- T cells in the acute stage and 6.29% in the regressive stage (Fig. 2B).

## 3 Discussion

In peripheral blood, CD4 negative CXCR5+ T cells that are recorded are within 1 subpopulation of CD8+ T cells. Although it has been more than a decade since the first description of CXCR5+CD8+ T cells,^[5]^ till recent years they are investigated as a new subset, which has been named follicular cytotoxic T (Tfc) cells.^[6]^ Accordingly, we would like to define those CD4-CXCR5+ T cells as Tfc-like cells. It has been documented that the percentage of circulating CXCR5+ T cells that express CD8 is less than 10% in healthy individuals.^[7]^ Furthermore, according to our previous studies that included 24 KD patients without coronary involvement and 20 healthy controls, the percentages of CD4 negative subpopulation were 6.45% (2.99–14.5) and 6.87% (2.6–10.3), respectively, of circulating CXCR5+ T cells.^[8]^ Thus, we believe that the levels of Tfc-like cells of the 2 patients in the acute stage are affirmatively increased.

Tfc cells that are increased during viral infection present an ability to eliminate infected cells.^[9,10]^ Furthermore, current understanding insists on the notion that KD is strictly relevant to viral infection.^[11,12]^ Hence, 1 possibility is that the increase of Tfc-like cells in our patients with CAA may result from a high viral load. However, there is no sufficient evidence to demonstrate that the formation of CAA is associated with viral load. Another scenario is that the increase of Tfc cells is associated with the function of cytotoxic immunity. At the early stage of infection, Tfc cells predominantly present a memory-like phenotype with a lower capacity to proliferate and perform cytosis in comparison with CXCR5-CD8+ T cells.^[10,13]^ With the persistence of the antigens, CXCR5-CD8+ T cells would gradually lose their cytotoxic capacity; in contrast, Tfc cells exhibit an enhanced ability of expansion and cytotoxin.^[9,10,13,14]^ In KD, histological biopsy from coronary artery of KD patients with CAA find infiltrated CD8+ T cells do not express perforin and granzymes,^[15]^ whereas the level of perforin+CD8+ T cells in the patients without CAA is comparable to that in healthy individuals, suggesting that the function of CD8+ T cells is seriously impaired in patients with CAA rather than the patients without CAA.^[16]^ Coincidentally, the level of Tfc-like cells is obviously increased in patients with CAA but not in those patients without CAA (according to our previous study). Therefore, we speculate that the increase of Tfc-like cells in the 2 patients is a compensatory result of the dysfunction of the cytotoxic immune response.

The invalid expression of the cytotoxic proteins of CD8+ T cells can lead to the persistence of causative agents and subsequently result in an uncontrolled inflammation, with finally the formation of CAA.^[15]^ Although the increase of Tfc-like cells is likely to be contributive for the clearance of pathogens, once increased Tfc-like cells are observed, maybe CAA has already formed, at least in our 2 patients. There have been studies that focus on the strategies to potentiate the function of Tfc cells to be against human immunodeficiency virus and simian immunodeficiency virus.^[17,18]^ Possibly, it also can be used in KD in advance to prevent the lost function of CD8+ T cells and thereby to restrain the formation of CAA. On the other hand, the pro-inflammatory cytokines produced by activated Tfc cells, such as IFN-γ,^[14]^ may induce the differentiation of macrophages towards a phagocytic phenotype^[19]^ and drive the infiltration of CXCR3 expressing cells into the vessel wall and subsequently aggravate the damage of coronary artery.^[20]^ Thus, the further studies that focus on their exact function in KD is needed. In summary, we first describe an increased level of Tfc-like cells in KD patients with CAA and speculate that Tfc cells are involved in the formation of CAA. The increase of Tfc-like cells is not only supportive of a relation between viral infection and KD, but also suggestive of a cytotoxic dysfunction of CD8+ T cells in KD patients with coronary aneurysms.

## Acknowledgments

We are very grateful to the 2 patients and their parents for their kind participation in this study. We thank the Key laboratory of Zoonoses Research of The First Hospital of Jilin University.

## Author contributions

**Conceptualization:** Meng Xu, Sirui Yang

**Data curation:** Jinxiang Liu, Lu pan

**Supervision:** Jinxiang Liu

**Writing – original draft:** Meng Xu

**Writing – review & editing:** Sirui Yang
